# Exploring the mechanism of andrographolide in the treatment of gastric cancer through network pharmacology and molecular docking

**DOI:** 10.1038/s41598-022-18319-0

**Published:** 2022-11-01

**Authors:** Ravi Prakash Yadav, Susanta Sadhukhan, Makhan Lal Saha, Sudakshina Ghosh, Madhusudan Das

**Affiliations:** 1 Department of Zoology, University of Calcutta, 35, Ballygunge Circular Road, Kolkata, 700 019 India; 2 Department of General Surgery, Institute of Post Graduate Medical Education & Research, Kolkata, 700020 India; 3 Department of Zoology, Vidyasagar College for Women, 39 Sankar Ghosh Lane, Kolkata, 700006 India

**Keywords:** Gene ontology, Virtual drug screening, Gastric cancer

## Abstract

Gastric cancer has emerged as a key challenge in oncology research as a malignant tumour with advanced stage detection. Apart from surgical management, a pharmacotherapeutic approach to stomach cancer treatment is an appealing option to consider. Andrographolide has been shown to have anticancer and chemosensitizer properties in a variety of solid tumors, including stomach cancer but the exact molecular mechanism is skeptical. In this study, we identified and validated pharmacological mechanism involved in the treatment of GC with integrated approach of network pharmacology and molecular docking. The targets of andrographolide and GC were obtained from databases. The intersected targets between andrographolide and GC-related genes were used to construct protein–protein interaction (PPI) network. Furthermore, mechanism of action of the targets was predicted by Gene Ontology (GO) and Kyoto Encyclopedia of Genes and Genomes (KEGG) pathway enrichment analyses. Finally, these results were validated by molecular docking experiments, mRNA and protein expression level. A total of 197 targets were obtained for andrographolide treating GC. Functional enrichment analysis revealed that the target genes were exerted promising therapeutic effects on GC by HIF-1 and PI3K-Akt signaling pathway. The possible mechanism of action is by inactivation of HIF-1 signaling pathway which is dependent on the inhibition of upstream PI3K-AKT pathway. The PPI network identified SRC, AKT1, TP53, STAT3, PIK3CA, MAPK1, MAPK3, VEGFA, JUN and HSP90AA1 as potential hub targets. In addition, these results were further validated with molecular docking experiments. Survival analysis indicated that the expression levels of the hub genes were significantly associated with the clinical prognosis of GC. This study provided a novel approach to reveal the therapeutic mechanisms of andrographolide on GC, making future clinical application of andrographolide in the treatment of GC.

## Introduction

Gastric cancer (GC) is the fifth most common cancer worldwide, with third highest mortality rate in the world^1^. In India, it is fifth most prevalent cancer in males and sixth most prevalent cancer in females with second most common cause of cancer mortality^2^. In spite of enormous research the development of GC is still not very clear^3^. Management of GC is remains challenging due to its poor prognosis and restricted treatment options^4^. Chemotherapy resistance and adverse side effects of drugs limit its clinical applications. The severe side effects of currently prescribed drugs include neutropenia, chemotherapy-induced peripheral neuropathy (CIPN), stomatitis, mucositis, diarrhoea, nausea, and emesis^5^. Therefore, the practice of complementary and alternative medicine gained economic and sociological importance as they are cost-effective and less toxic^6^. These alternative medicinal dietary compounds are not harmful and extracted from plants. Most of these botanical compounds exert their effect on regulation of cancer-associated pathways to prevent tumorigenesis and as well as cancer progression^7^. Among the alternative medicinal compounds, Andrographolide get attention for the treatment of GC because of its anti-inflammatory, antiviral, immunomodulatory^8,9^ and anti-tumorigenic role in melanoma, leukemia, glioblastoma, breast, lung, esophageal, colorectal, bladder, pancreatic, and liver cancer^10–19^. Andrographolide is a traditional Chinese herbal medicine isolated from *Andrographis paniculate*^20^ and have the ability to circulate in the bloodstream^21^. Furthermore, previous studies have reported potential role of andrographolide in regulation of oxidative stress, apoptosis, necrosis, autophagy, inhibition of cell adhesion, proliferation, migration, invasion, and angiogenesis^10,20,22,23^. Various cancer-related and angiogenesis signaling pathways, such as PI3K/AKT/mTOR^11,15^ SRC/MAPKs/AP-1^24^, TLR4/NF-κB/MMP-9^16^, and VEGF/VEGFR2/AKT^19^ are also controlled by andrographolide. In recent, several studies reported that in human cancer cells andrographolide treatment promotes the rate of apoptosis and inhibit cell proliferation. Similarly, it is also evident for GC cells, apoptosis guided by tumor necrosis factor-related apoptosis-inducing ligand (TRAIL) controlled by andrographolide^12^, but, in GC, the mechanistic pathways of anti-tumorigenic actions mediated by andrographolide are still unknown.

So, the main aim of the present study is to investigate the pharmacological mechanisms of andrographolide in treating gastric cancer using network pharmacology and molecular docking experiments.

## Materials and methods

### Drug-likeness prediction

**Figure 1:**
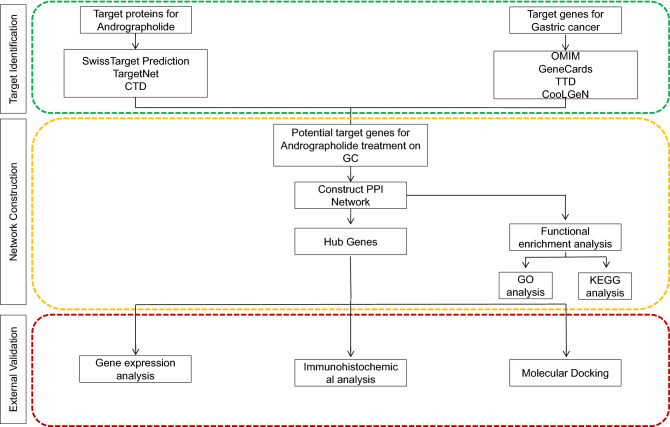
General workflow of network pharmacology and molecular docking in the present study.

Drug-likeness was evaluated by following Lipinski’s rule of five (RO5) for screening potential oral drugs in humans. The parameters evaluated are molecular weight (MW), XLogP3 (octanol–water partition coefficient), topological polar surface area, number of rotatable bonds, hydrogen bond acceptor, and hydrogen bond donor numbers. To explore the drug-likeness properties of andrographolide, the SMILES format CC12CCC(C(C1CCC(=C)C2CC=C3C(COC3=O)O)(C)CO)O of andrographolide was uploaded into the SwissADME server (http://www.swissadme.ch), an online tool for calculating the absorption, distribution, metabolism, excretion (ADME), oral bioavailability (OB), and drug-likeness (DL)^25^. The overall flowchart of this study is shown in Fig. 1.

### Target proteins of andrographolide

The SwissTargetPrediction databases (http://www.swisstargetprediction.ch/)^26^, the TargetNet (http://targetnet.scbdd.com/)^27^ and the Comparative Toxicogenomics Database (CTD, http://ctdbase.org/)^28^ were used to predict the targets of andrographolide. The 3D molecular structure file and the canonical SMILES of andrographolide were imported into the SwissTargetPrediction, TargetNet and CTD databases, respectively. Further, the predicted candidate targets were normalized by UniProt database (http://www.uniprot.org/).

### Potential targets in GC

To ensure a comprehensive collection of GC-related genes four online public database sources were explored and GC-related genes were downloaded, including the Online Mendelian Inheritance in Man (OMIM) database (http://www.omim.org), GeneCards database (https://www.genecards.org/), Therapeutic Target Database (TTD, http://bidd.nus.edu.sg/group/cjttd/)^29^, and CooLGeN database (http://ci.smu.edu.cn/CooLGeN/)^30^. Targets with a hit score greater than 5 in the CooLGeN database were chosen as GC-related genes. Then, the predicted targets of the andrographolide were mapped with the targets of GC, and the intersection of the two was taken to obtain the target set of andrographolide for the treatment of GC.

### Construction of compound-target network

Andrographolide and its therapeutic targets in GC were introduced into Cytoscape (https://cytoscape.org/; Version 3.8.2) to construct the compound-target network^31^.

### Gene Ontology and pathway enrichment

Gene Ontology (GO) and Kyoto Encyclopedia of Genes and Genomes (KEGG) pathway enrichment analysis were performed with the Database for Annotation, Visualization and Integrated Discovery (DAVID, https://david.ncifcrf.gov/, ver. 6.8). KEGG is pathway database consisting of graphical diagrams of biochemical pathways^32^. The Gene Ontology (GO) is a comprehensive resource for functional genomics that includes gene function definitions^33^. Based on pathological and clinical data pathways related to GC were selected. Histograms and bubble charts are produced through the Bioinformatics cloud platform (http://www.bioinformatics.com.cn/, an online platform for data analysis and visualization).

### Protein–protein interaction analysis

Protein–protein interaction (PPI) plays a significant role in biological processes and is essential for understanding the intricate systems that operate within a living cell^34^. PPI network mapping was performed on the cluster of target genes using the Search Tool for the Retrieval of Interacting Genes database (http://string-db.org/; version 11.5) with the species limited to “Homo sapiens” and a confidence score of > 0.9 to ensure high confidence information. The PPI network was constructed by Cytoscape (https://cytoscape.org/; version 3.8.2), a bioinformatics software used for data visualization and integration^31^. To find clusters (highly interconnected regions) within the PPI network, the Cytoscape plugin cytoHubba (https://apps.cytoscape.org/apps/cytohubba; version 0.1) version 0.1) was used. Based on the degree level, the top-ranked proteins were designated as hub targets. We identified the protein clusters using MCODE^35^, a Cytoscape plug-in. Then, proteins contained in each cluster were entered into the STRING database to reproduce the corresponding cluster map and predict functional associations between proteins.

### Molecular docking analysis between andrographolide and hub genes

To gain an in-depth insight into the relationship and action mechanisms between candidate proteins and andrographolide, molecular docking was conducted to assess the strength and mode of interactions between andrographolide and the hub targets. The molecular docking simulation was conducted by CB-Dock (http://cao.labshare.cn/cb-dock. It can automatically identify the active sites of a given protein, calculate the center and size, customize the grid box size according to the query ligands, and then perform molecular docking with a popular docking program, AutoDock Vina^36^. The crystal structures of the hub target proteins were downloaded from the protein data bank (http://www.rcsb.org). The 3D structure of andrographolide was downloaded from the PubChem compound database (https://pubchem.ncbi.nlm.nih.gov/; PubChem ID-5318517). Next, the crystal structures of proteins and the ligand file of andrographolide were inputted to CB-Dock, and docking analysis was conducted to elucidate the binding activities. The docked results were then visualized and analyzed using Discovery Studio Visualizer software (Accelrys Software Inc.)^37^.

### External validation of hub genes

#### Gene expression levels of hub gens

Gene Expression Profiling Interactive Analysis (http://gepia2.cancer-pku.cn/) is an online server providing interactive and customizable functions based on the Cancer Genome Atlas (TCGA) and Genotype-Tissue Expression (GTEx) database. GEPIA was used to validate the differential expression of the hub genes between GC and normal gastric tissues, and it can also analyze them according to pathological stages^38^.

#### Protein expression levels of hub genes

The Human Protein Atlas (Version 20.1) (HPA, https://www.proteinatlas.org/) database is an extensive proteome database based on immunohistochemical analysis. According to the intensity of staining and percentage of stained cells in the tissues, the protein expression levels of hub genes in GC tissues and normal gastric tissues were compared, and representative immunohistochemical staining pictures were obtained from the database HPA^39^.

#### Overall survival analysis of hub genes

To explore the hub targets’ influence on the overall survival (OS) of GC, a cancer genomics dataset named Kaplan–Meier Plotter (http://kmplot.com/analysis/index.php?p=service)^40^, which is capable of assessing the effect of genes on survival, was used to estimate the prognostic significance of each hub gene. The patients with GC were separated into two groups: those with high expression and those with low expression, and a Kaplan–Meier survival plot was used to compare the two groups. The hazard ratio (HR) with 95% confidence intervals and the log rank P value were calculated.

## Results

### Molecular properties of andrographolide

Predicting for potential therapeutic ligands is the major outcome of Drug-likeness assessment. Our results showed that the property of andrographolide is according to Lipinski’s RO5. The polar surface area (PSA) less than or equal to 140 A^2^ whereas MW of a drug-like compound is < 500 g/mol and calculated octanol/- water partition coefficient (XLogP3) < 5, these 3 are the most important factors for drug ligand interactions. On the other hand, the rotatable bond less than 10, the hydrogen bond acceptors no more than 10, and the hydrogen bond donors no more than 5. Molecular properties of andrographolide were shown in Table 1, which are in line with the RO5, indicating that it had good drug-like properties.

**Table 1:** Molecular properties of andrographolide.

Property	Value
Molecular weight	350.45 g/mol
PSA 131.36	86.99 A2
XLogP3	2.16
Rotatable bonds	3
H-bond donor	3
H-bond acceptor	5
Molar refractivity	95.21
Bioavailability score	0.55

### Target identification and analysis

**Figure 2:**
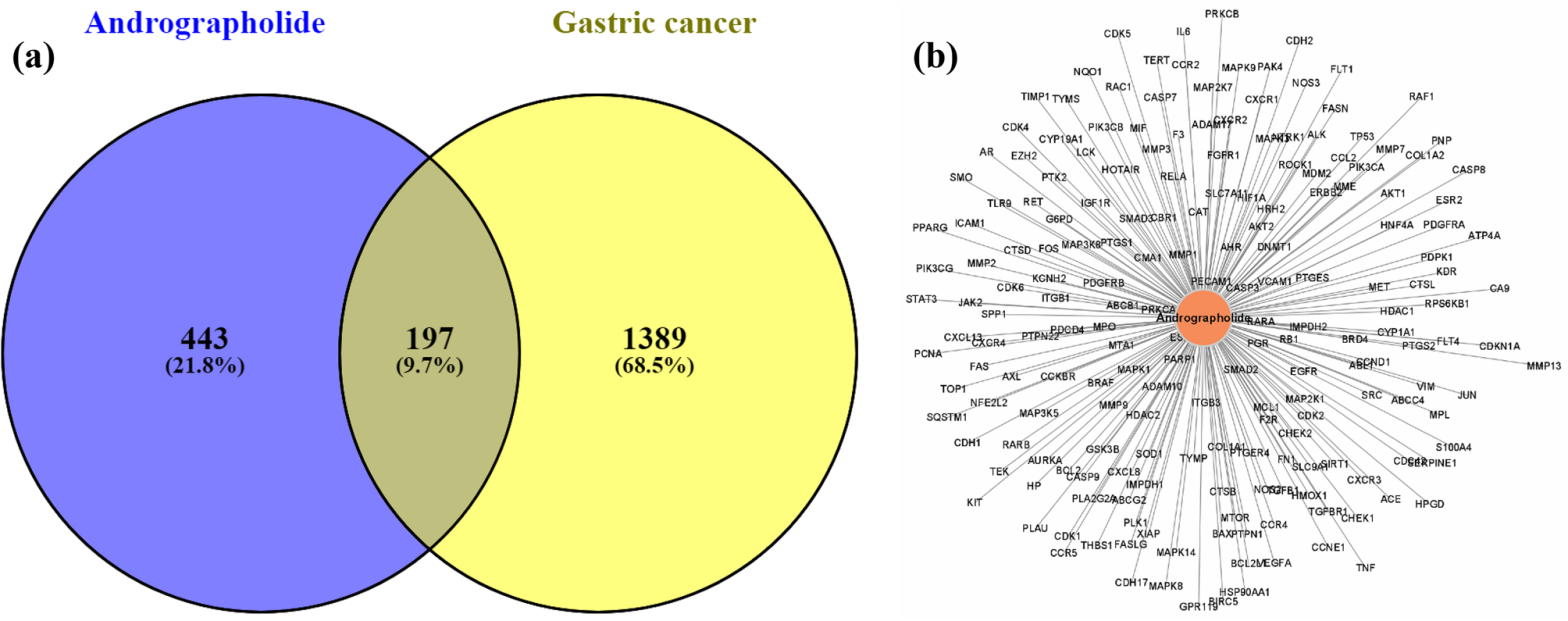
Common-target network. (**a**) Venn diagram of the intersection relationship of target genes between Andrographolide and GC. (**b**) Compound-Target Network generated using Cytoscape (https://cytoscape.org/; ver. 3.8.2).

OMIM, GeneCards, TTD, and CooLGeN databases were screened with the search term gastric cancer and stomach cancer, to determine GC-related targets. A total of 1586 targets related to GC were identified. Furthermore, targets for andrographolide were searched in SwissTargetPrediction, CTD and TargetNet databases and 640 targets were identified. A comparison between andrographolide and GC related targets identified 197 intersection genes. Figure 2 shows the compound-target network (C-T network) consisting of 198 nodes and 197 edges suggesting the role of andrographolide in regulation of these genes for GC treatment.

### GO enrichment analysis

**Figure 3:**
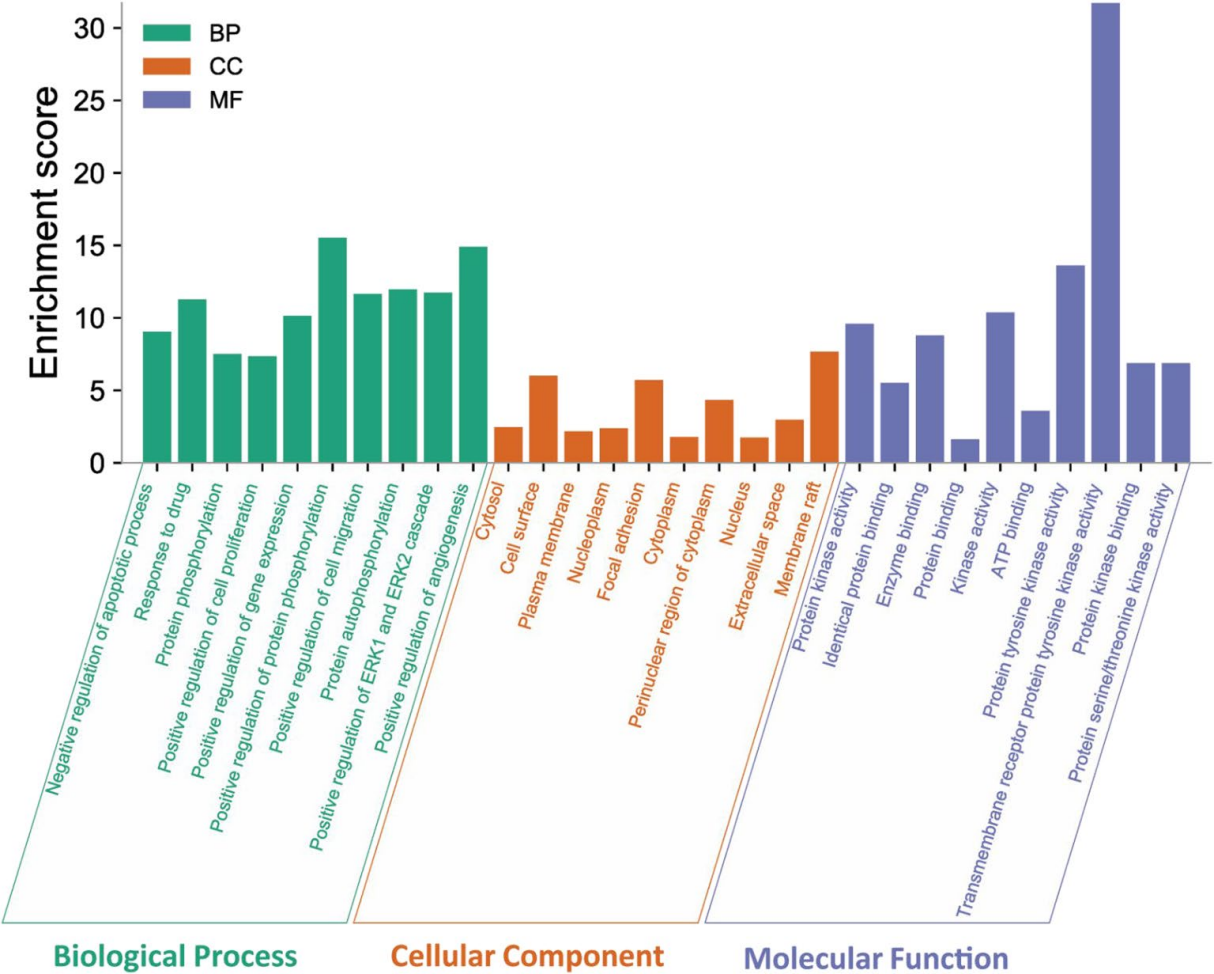
GO enrichment analysis for the targets in andrographolide treating gastric cancer (P value < 0.05) (http://www.bioinformatics.com.cn/).

GO enrichment analysis of the 197 targets, identified a total of 809 GO terms. Figure 3 shows the results of the GO Enrichment. The biological process (BP) results suggest that these targets regulate apoptosis, angiogenesis and ERK1/ERK2 cascade, gene expression, drug metabolism and oxidative stress. Component cellular (CC) results included the cell surface, focal adhesion and membrane raft. For molecular function (MF), the targets mostly involved in the kinase activity.

### KEGG enrichment analysis

**Figure 4:**
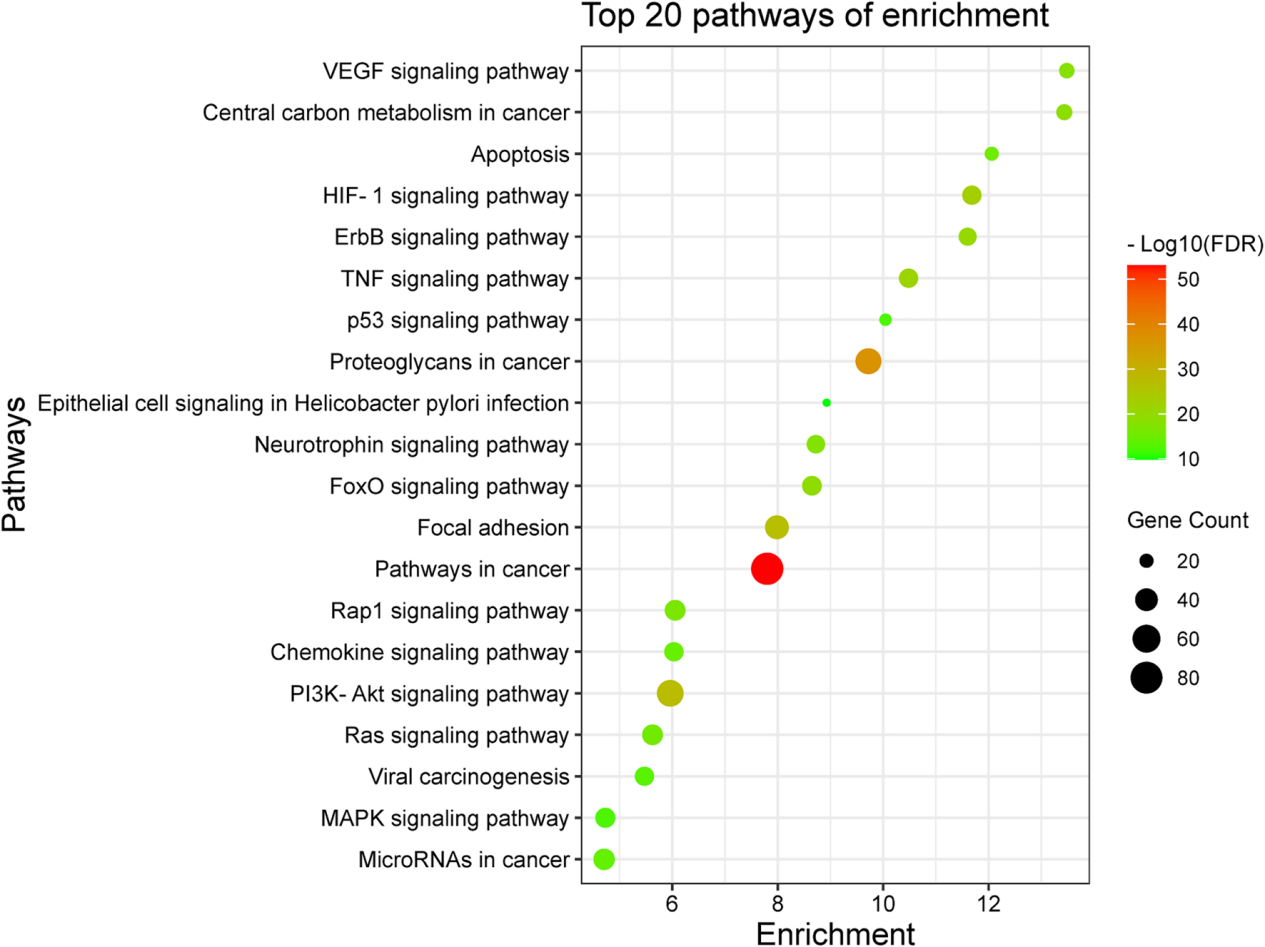
The top 20 potential KEGG pathway enrichment of screened target genes in GC (http://www.bioinformatics.com.cn/).

A total of 105 pathways were identified by KEGG pathway analysis, and the targets were closely related to pathways in cancer, PI3K-Akt signaling pathway, Proteoglycans in cancer, Focal adhesion, microRNAs in cancer, Rap1 signaling pathway, Ras signaling pathway, MAPK signaling pathway, FoxO signaling pathway and HIF-1 signaling pathway. This shows that andrographolide may play a role in the treatment of GC through the above-mentioned pathways, among which the PI3K signaling pathway is the key pathway involving 55 potential targets, including most of the hub genes. According to the number of enriched genes, the top 20 remarkably enriched pathways were shown as likely to be the major pathways in the treatment of GC (results can be seen in Fig. 4).

### Construction of protein–protein interaction network (PPI) and identification of key targets

**Figure 5:**
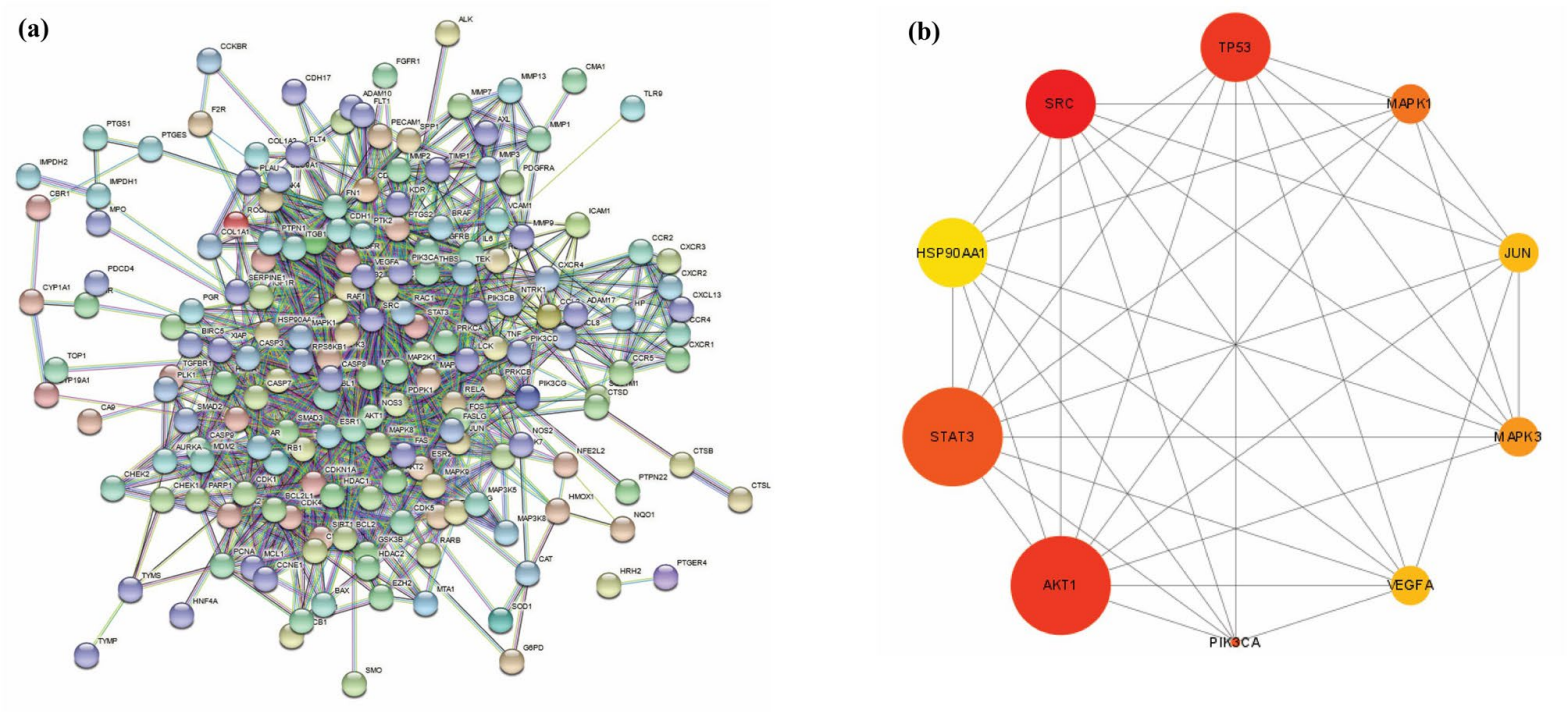
Protein–protein interaction (PPI) network and Hub gene analysis. (**a**) PPI network performed by STRING database. Contains 197 common DEGs. (http://string-db.org/; version 11.5). (**b**) PPI network of the top 10 hub genes generated by (Cytoscape plugin cytoHubba; https://apps.cytoscape.org/apps/cytohubba; version 0.1) (the redder the colour, the more important it is).

The common targets of andrographolide and GC were put into STRING database for PPI network construction (results can be seen in Fig. 5a). Furthermore, using cytoHubba plugin in Cytoscape, topological analysis was carried by degree selection. Hub genes were selected based on their degree value and considered as key regulator genes of treatment of GC (results can be seen in Fig. 5b). The darker the color, the higher the degree^41^. Cluster analysis was performed using MCODE plugin. Four clusters were identified with cluster 1, contained 28 nodes and 108 edges, including SRC, STAT3 and TP53. Cluster 2 contained 32 nodes (VEGFA, MAPK3, MAPK1 and HSP90AA1) and 124 edges. Cluster 3 contained 5 nodes 10 and cluster 4 contained 11 nodes and 21 edges, respectively and were listed in Table 2.

**Table 2:** Clusters of the protein–protein interaction (PPI) network.

Cluster	Score	Nodes	Edges	Gene IDs
1	8	28	108	CDKN1A, PCNA, SRC, STAT3, CCR2, CXCL8, FOS, CCR5, FN1, KDR, CDK1, CDK2, CXCR1, CDK6, CXCR3, CCNE1, CCR4, MAP2K1, CXCR4, CXCR2, CDK4, CXCL13, CCND1, ABL1, ERBB2, BRAF, TP53, ITGB1
2	8	32	124	MTA1, CDC42, MET, EGFR, VEGFA, ITGB3, MAPK3, PAK4, HIF1A, RAC1, PTK2, FLT1, BCL2, LCK, AKT2, TERT, PIK3CD, RPS6KB1, MAPK1, PPARG, PDGFRB, PIK3CG, HSP90AA1, JAK2, PRKCA, SMAD3, MAPK8, CDH1, EZH2, FLT4, HDAC2, BCL2L1
3	5	5	10	MMP7, MMP2, MMP3, MMP13, MMP1
4	4.2	11	21	PIK3CB, IGF1R, SIRT1, AKT1, RAF1, PDGFRA, PIK3CA, MAPK14, TNF, RB1, JUN

### Confirmation of hub target by molecular docking

**Figure 6:**
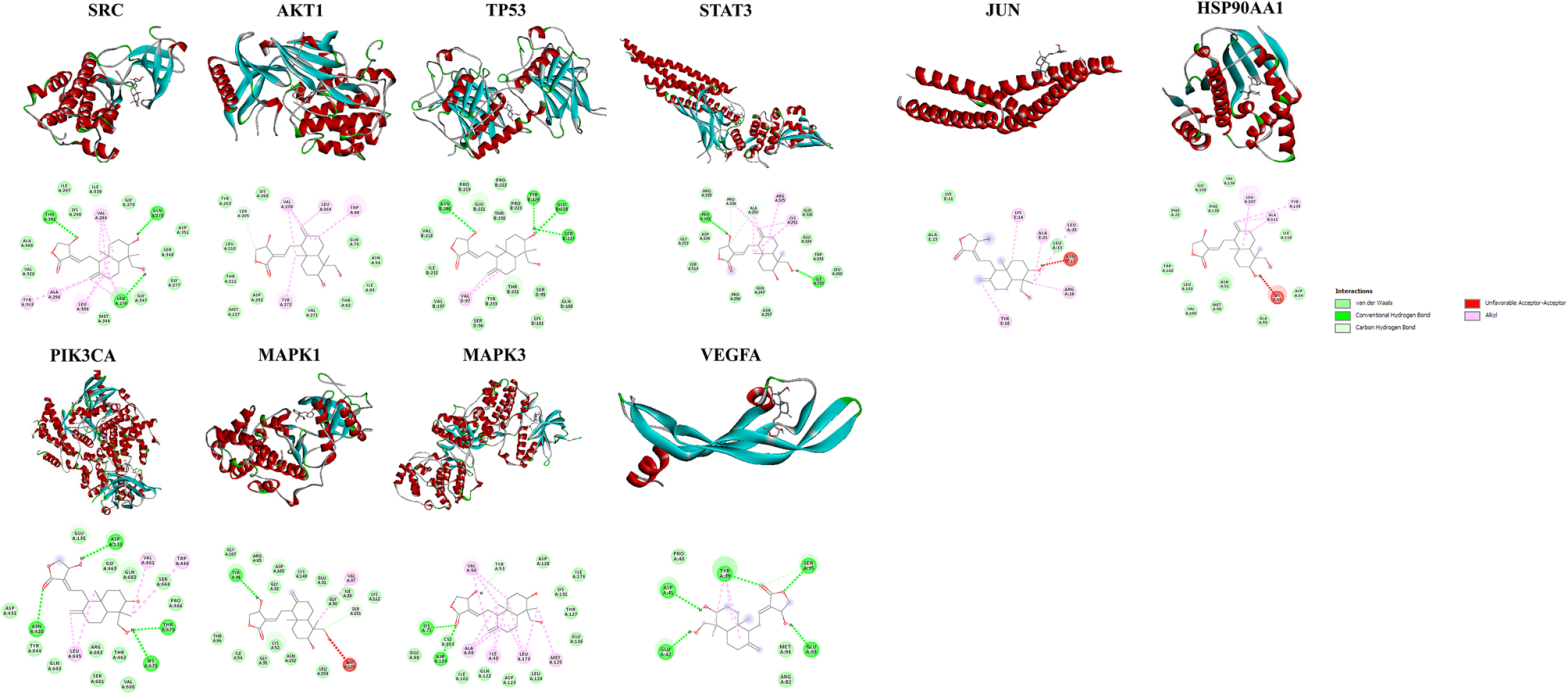
Molecular docking results of hub targets with andrographolide. (https://discover.3ds.com/discovery-studio-visualizer-download).

To verify the reliability of the drug-target interactions, the ten hub proteins were selected as targets for molecular docking. The structure of andrographolide was uploaded to CB-DOCK for analysis of docking potential with SRC, AKT1, TP53, STAT3, PIK3CA, MAPK1, MAPK3, VEGFA, JUN and HSP90AA1. It is generally believed that the lower the energy when the conformation of the ligand binding to the receptor is stable, the greater the possibility of action. In this study, all core target proteins’ and andrographolide binding energies were less than − 5.0, which indicated that andrographolide had better binding activity with core targets (Table 3). All the binding energies were listed in Table 3 and all the docking sketch maps of the target protein with andrographolide are shown in Fig. 6.

**Table 3:** Molecular docking scores of andrographolide and hub target proteins.

Receptor	PDB ID	Binding energy kcal/Mol
SRC	6ATE	− 7.8
AKT1	6S9X	− 9.2
TP53	3Q05	− 7.8
STAT3	6NJS	− 7
PIK3CA	5DXT	− 8.7
MAPK1	3O71	− 7
MAPK3	4QTB	− 8.9
VEGFA	4KZN	− 5.7
JUN	5FV8	− 6
HSP90AA1	4BQG	− 7.1

### External validation of hub genes

#### mRNA expression levels of hub gens

**Figure 7:**
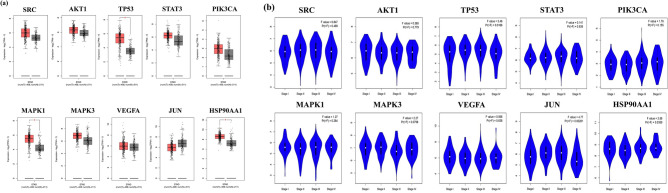
The mRNA expression levels of hub genes in The Cancer Genome Atlas (TCGA) and Genotype–Tissue Expression (GTEx) databases. (**a**) Boxplot of hub gene mRNA expression levels in the GEPIA database. Red represents GC tissue, and grey represents normal gastric tissue. (**b**) Stage plot of hub genes mRNA expression level and pathological stage in the GEPIA database.

We used the GEPIA database to analyze the differential expression of hub genes between GC tissues and normal tissue. Tp53, MAPK1, and HSP90AA1 mRNA levels were considerably higher in GC specimens than in normal gastric samples (P < 0.01) (results can be seen in Fig. 7a). In addition, we analyzed the relationship between hub gene mRNA levels and the pathological stages of GC. The results showed that the levels of TP53, STAT3, JUN and HSP90AA1 changed significantly with pathological stage and increased significantly in stage III (results can be seen in Fig. 7b). These findings suggested that these four genes' expression levels might be linked to GC advancement.

#### Protein expression levels of hub gens

**Figure 8:**
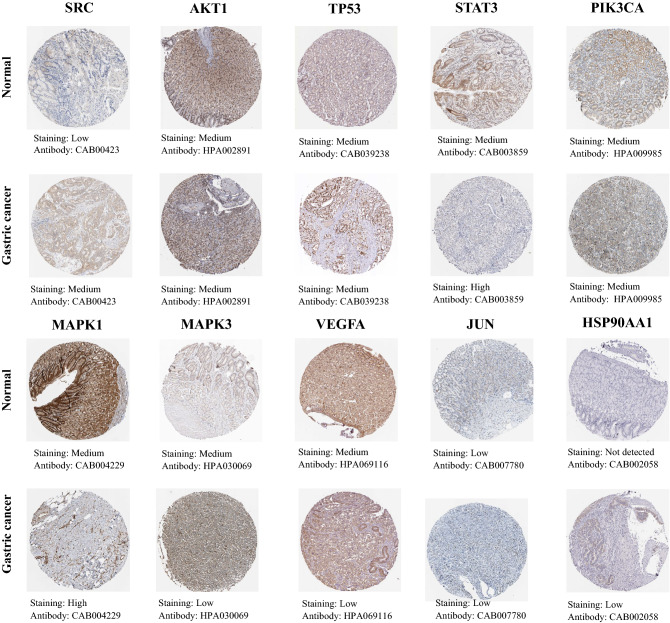
Immunohistochemical images of hub gene protein expression levels in the HPA database.

Additionally, we analyzed the immunohistochemical staining images in the HPA database to observe the expression levels of hub gene proteins in GC. Except for HSP90AA1, the other nine hub genes were expressed to varying degrees in normal stomach tissues. The expression levels of SRC, STAT3, MAPK1 and HSP90AA1 were increased in GC tissues, while the expression of MAPK3 and VEGFA were decreased in GC tissues in comparison to normal gastric tissues (results can be seen in Fig. 8).

#### Survival analysis of the hub genes

**Figure 9:**
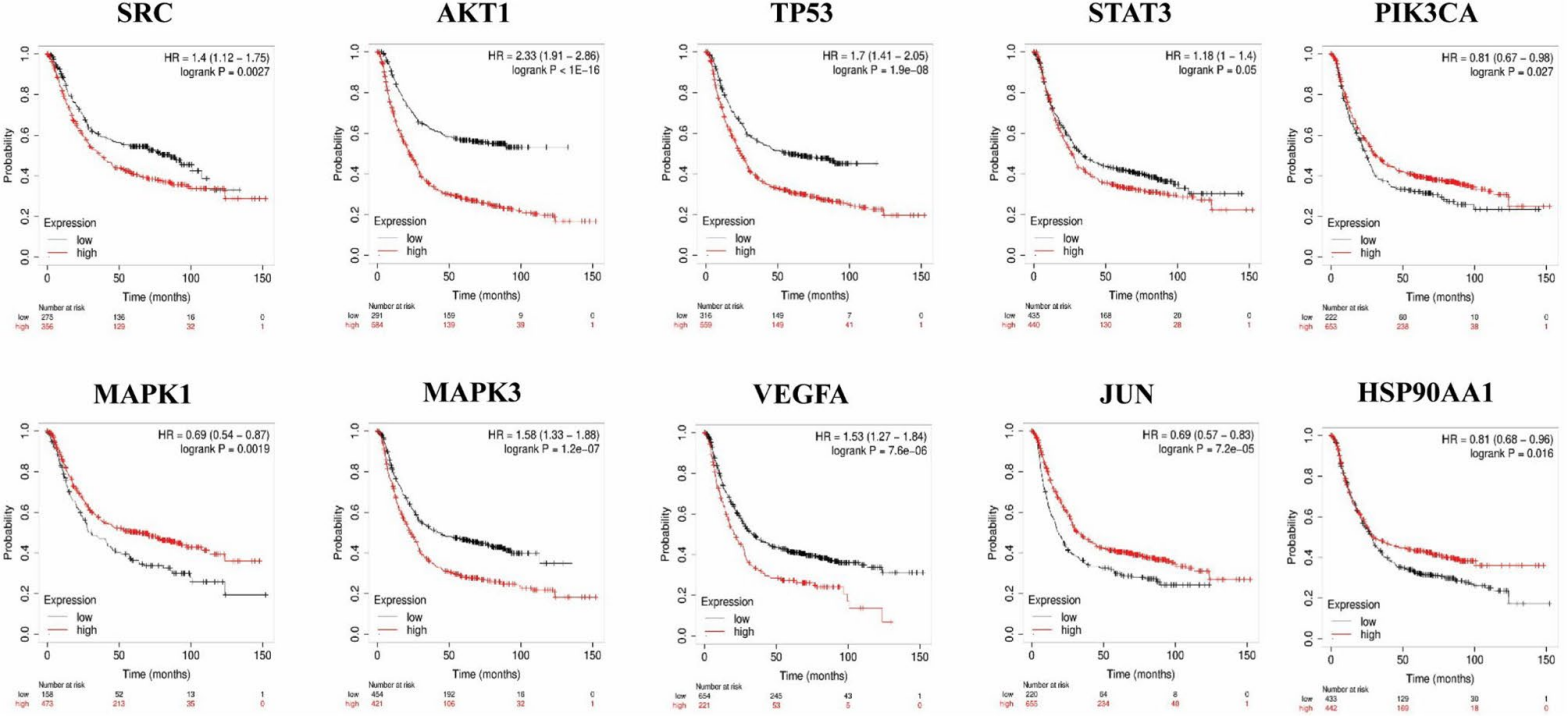
Kaplan–Meier overall survival analyses of patients with gastric cancer based on expression of the ten hub genes. HR, hazard ratio (http://kmplot.com/analysis/index.php?p=service).

Survival plots were performed for 10 hub genes including SRC, AKT1. TP53, STAT3, PIK3CA, MAPK1, MAPK3, VEGFA, JUN and HSP90AA1. The results suggested that all the hub genes significantly associated with the poor prognosis of 875 GC patients from TCGA database (P < 0.05, results can be seen in Fig. 9).

## Discussion

The incidence of GC is extremely high in many countries worldwide, and much higher in Eastern Asia^42^. Andrographolide, a natural compound from plants, has been reported to exert anti-GC activity via various mechanisms. The effect of andrographolide against GC has been studied previously^43^, however, the exact molecular mechanisms of andrographolide against GC are still not completely understood.

For complex diseases like GC, network pharmacology has widely used in many studies unique for predictive analysis^29^. In this study, a network pharmacology approach integrating drug-likeness evaluation, target identification, GO and pathway analyses, and PPI analysis was successfully established to systematically analyze the potential molecular mechanism of andrographolide in treating GC.

SRC, AKT1. TP53, STAT3, PIK3CA, MAPK1, MAPK3, VEGFA, JUN and HSP90AA1 are the hub genes which are key targets of andrographolide for the treatment of GC. Andrographolide targets the pathways involved in cancer, PI3K-Akt signaling and HIF signaling pathways. Previous studies have shown that andrographolide decreased cell invasion by PI3K-Akt-mTOR signaling pathway^44^ and suppressed cell migration through Hif1α and PI3K-Akt signaling pathway^45^.

The mechanisms behind effects of andrographolide was broadly through inhibition of v-Src, NF-κB, STAT3 and PI3K/AKT activity and downregulation of mediators of cell cycle progression, inflammation, metastasis and angiogenesis^45^. Several studies have proven that andrographolide can attenuate cancer cell migration and invasion by suppressing the protein levels of Akt1^20^. Frequent activation of AKT has also been reported in GC and other cancers^46^. Approximately, in 50% GC cases TP53 (tumor protein p53) is mutated and is most commonly mutated gene in human cancers. This gene plays significant roles in cell cycle arrest, cellular senescence, apoptosis, differentiation and metabolism^47^. STAT3 acts as a carcinogen in GC, which can enhance the metastatic potential of tumor cells and promote the development and progression of tumors^48^. Andrographolide induces apoptosis and enhances anticancer activity through inactivating STAT3 and Akt^49^. Andrographolide can be combined with conventional chemotherapeutic agents to be used as a potential therapeutic strategy. It enhanced chemosensitivity of tumor cells to doxorubicin through inhibition of STAT3 activity^50^. Andrographolide inhibited the PI3K expression and phospho-Akt coexisting with cell invasion and migration mechanism^51^. MAPK is a crucial signal transmitter in cells that is involved in a variety of biological processes such as cell proliferation, differentiation, and immune defense by phosphorylating nuclear transcription factors and related enzymes. The abnormal expression of MAPK3 was related to the invasion, metastasis, and drug resistance of a variety of tumor cells. Kim et al. found that MAPK3 expression is an independent prognostic index for patients after gastrectomy^52^. It has been reported that andrographolide has anti-angiogenic activity which is mediated through preventing VEGFA-induced phosphorylation and activation of VEGFR2 and MAPKs^53^. Transcription factor JUN is a risk gene for GC, and it can promote the occurrence and development of GC by participating in the regulation of the MAPK signaling pathway^54^. Andrographolide that involved inhibiting Hsp90 function and downregulating Hsp90 client proteins, broadening the molecular basis of andrographolide-mediated anticancer effects^55^.

All these results suggest that andrographolide can be considered as prospective anticancer and chemosensitizer with the ability to overcome resistance to chemotherapy drugs in gastric cancer cells^6^. Taken together, SRC, AKT1. TP53, STAT3, PIK3CA, MAPK1, MAPK3, VEGFA, JUN and HSP90AA1 are crucial in the pathogenesis of GC. Hence, it is promising to discover more novel anticancer compounds with low toxicity based on network pharmacology analysis. This study provides a rationale for using andrographolide for the treatment of GC.

In our study we found that andrographolide is a multitarget anticancer drug. We predicted that the primary mechanism of action of andrographolide in the treatment of GC is mediated by HIF-1, PI3K-AKT and other signaling pathways to regulate the proliferation, apoptosis, migration, and angiogenesis of tumour cells, thus playing a role in the treatment of GC.

## Conclusion

In this study, based on network pharmacology analysis, we predicted 10 potential center targets for andrographolide in the treatment of GC, suggesting that andrographolide can be an herbal medicine with multicomponent, multiple targets, and multiple pathways. The network analysis revealed that andrographolide may exert its therapeutic effects against GC by modulating certain distinct targets, such as SRC, AKT1. TP53, STAT3, PIK3CA, MAPK1, MAPK3, VEGFA, JUN and HSP90AA1. The GO analysis of these targets results showed that andrographolide likely produced pharmacological effects against GC mainly by influencing different biological processes, like negative regulation of apoptotic process, response to drug, positive regulation of cell proliferation, migration, and angiogenesis. Meanwhile, the KEGG pathway analysis in the present study disclosed that andrographolide probably exerted its pharmacological action via simultaneously regulating different signaling pathways related to GC, such as pathways in cancer, HIF-1 signaling pathway and PI3K-AKT signaling pathway. These findings supported the possibility that andrographolide mediates HIF-1 inhibition which may be dependent on the inhibition of upstream PI3K-AKT pathway.

To summarize, andrographolide is a promising compound, which is expected to be developed as a safe and effective multitarget drug against GC. Our network pharmacological analysis predicted that andrographolide may exert an anti-GC effect through multiple targets, pathways, and biological processes, thereby regulating the cell metabolism and cell apoptosis. Further verification studies are required to confirm the clinical efficacy of andrographolide and its mechanisms against GC.

## Data Availability

All data in this paper can be collated from the open-source website provided by us and analyzed by relevant software.
